# Bubonocele in Horse

**Published:** 1890-12

**Authors:** S. D. Brimhall


### BUBONOCELE IN HORSE.

By S. D. Brimhall, V.M.D.

A chestnut stallion about sixteen hands high, seven years old, became mired while working on soft ground ; that night he had slight colic pains, but worked for a week without trouble when he again showed signs of pain. Two days later the owner noticed a swelling of the sheath and scrotum, and called a quack, who said the horse's kidneys were all wrong, etc. Four days later I was called by the owner to treat the case, and found the animal eating and drinking well ; temperature, 102° ; pulse, 52 ; urine and fæces passed regularly. There was an enormous swelling of left scrotal region and the sheath, which were hot and painful to touch.

Upon rectal examination I found the left inguinal ring completely closed by a hernia of a portion of the small intestines, which had found firm adhesion and was fast shutting off the circulation of the cord. As all that could be done at this time was to assist nature, I endeavored to do so by the use of laxatives and cold applications, with support to the scrotum, but the swelling was so great that in a few days the scrotum became cold, and a large piece sloughed away, leaving the atrophied testicle exposed ; at this time, while examining the condition of the cord, I removed a small amount of fæces which appeared quite fresh ; in a few days the testicle and protruding portion of cord sloughed away, fæces were passed through this opening for about four weeks, when the wound had healed so that there only remained a narrow fistula through which passed a small amount of serous fluid.

